# Genome evolution driven by host adaptations results in a more virulent and antimicrobial-resistant *Streptococcus pneumoniae *serotype 14

**DOI:** 10.1186/1471-2164-10-158

**Published:** 2009-04-13

**Authors:** Feng Ding, Petrus Tang, Mei-Hua Hsu, Peng Cui, Songnian Hu, Jun Yu, Cheng-Hsun Chiu

**Affiliations:** 1 The CAS Key Laboratory of Genome Sciences and Information, Beijing Institute of Genomics, Chinese Academy of Sciences, 100029 Beijing, PR China; 2 Graduate School of the Chinese Academy of Sciences, Chinese Academy of Sciences, 100029 Beijing, PR China; 3 Department of Parasitology, Chang Gung University College of Medicine, Kweishan 333, Taoyuan, Taiwan; 4 Department of Pediatrics, Chang Gung Children's Hospital, Chang Gung University College of Medicine, Kweishan 333, Taoyuan, Taiwan

## Abstract

**Background:**

*Streptococcus pneumoniae *serotype 14 is one of the most common pneumococcal serotypes that cause invasive pneumococcal diseases worldwide. Serotype 14 often expresses resistance to a variety of antimicrobial agents, resulting in difficulties in treatment. To gain insight into the evolution of virulence and antimicrobial resistance traits in *S. pneumoniae *from the genome level, we sequenced the entire genome of a serotype 14 isolate (CGSP14), and carried out comprehensive comparison with other pneumococcal genomes. Multiple serotype 14 clinical isolates were also genotyped by multilocus sequence typing (MLST).

**Results:**

Comparative genomic analysis revealed that the CGSP14 acquired a number of new genes by horizontal gene transfer (HGT), most of which were associated with virulence and antimicrobial resistance and clustered in mobile genetic elements. The most remarkable feature is the acquisition of two conjugative transposons and one resistance island encoding eight resistance genes. Results of MLST suggested that the major driving force for the genome evolution is the environmental drug pressure.

**Conclusion:**

The genome sequence of *S. pneumoniae *serotype 14 shows a bacterium with rapid adaptations to its lifecycle in human community. These include a versatile genome content, with a wide range of mobile elements, and chromosomal rearrangement; the latter re-balanced the genome after events of HGT.

## Background

*Streptococcus pneumoniae *is a major human respiratory pathogen that causes a variety of serious infections such as pneumonia, otitis media, meningitis and hemolytic uremic syndrome (HUS). It is estimated that each year more than 1 million deaths are attributed to *S. pneumoniae *infection worldwide [1]. Furthermore, since 1990 antimicrobial resistance has been escalating in *S. pneumoniae *[2], resulting in difficulties in the treatment of pneumococcal infections. Antimicrobial resistance of *S. pneumoniae *is associated with increasing incidence of invasive pneumococcal diseases in children as well as clinical failures of antimicrobial treatment [3]. Although the 7-valent conjugate vaccine has been shown highly efficacious in the prevention of invasive diseases caused by vaccine serotypes [4], the emergence of non-vaccine serotype infections has occurred [5], bringing new challenges to the prevention of pneumococcal diseases.

HUS is a rare but severe complication of infectious diseases. The majority of HUS cases are associated with enterohemorrhagic *Escherichia coli*; however, HUS associated with invasive *S. pneumoniae *infection has been increasingly reported over the years, always with a high mortality and long-term morbidity [6]. Among the vaccine-preventable serotypes of *S. pneumoniae*, serotype 14 is often invasive, as evidenced by its high predilection to cause necrotizing pneumonia and other devastating complications, including HUS [7,8]. In the pre-vaccine era, serotype 14 was one of the most common serotypes of *S. pneumoniae *that caused invasive pneumococcal diseases worldwide [9-11]. Moreover, serotype 14 often expresses resistance to a variety of antimicrobial agents, including penicillin, erythromycin, and ceftriaxone. To date, four complete genome sequences of *S. pneumoniae *are available in public database, including two laboratory strains (TIGR4 and D39), one avirulent strain (R6) and one multidrug-resistant strain (Spn23F) [12-15]. Furthermore, a total of twelve draft sequences have been published recently [16,17]. These pneumococcal genomes provide us a good opportunity to undertake comprehensive comparative studies on the virulence and antimicrobial resistance mechanisms of this microorganism. We described here the features of *S. pneumoniae *serotype 14 genome and the changes of the genome structure and contents compared with other pneumococcal genomes. The diversity and dynamics of the distributed genome of *S. pneumoniae *serotype 14, especially the mobile genetic elements carrying a number of virulence genes and antimicrobial resistance determinants, are highlighted.

## Results

### General genome features

**Figure 1:**
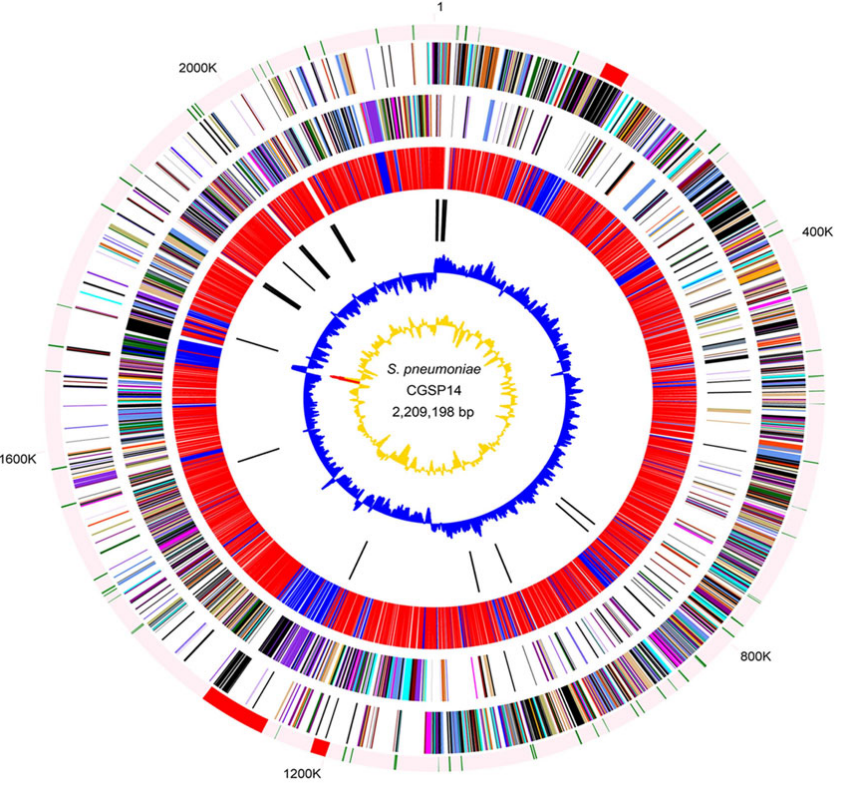
**Circular representation of *S. pneumoniae *chromosome**. Outer circle: distribution of mobile genetic elements (red, transposons and putative resistance islands; green, IS elements). Second and third circles: predicted ORFs on the plus and minus strand, respectively. ORFs are distinguished by different colors in the COG classification . Fourth circle: conserved genes (red) of the four *S. pneumoniae *genomes and accessory elements (blue). Fifth circle: tRNA and rRNA genes. Sixth circle: GC skew. Seventh circle: GC content.

The single circular chromosome of strain CGSP14 contains 2,209,198 bp with a G + C content of 39.5% (Figure 1). The sequence of the genome has been deposited in the GenBank database (accession no. CP001033). Base pair one of this chromosome was assigned within the putative origin of replication. The genome has 58 tRNAs, 12 rRNAs and 3 structural RNAs, including 4 rRNA operons. Biological roles were assigned to 67% of the 2,206 predicted protein-coding sequences (CDSs), according to the classification scheme adapted from Riley [18]. Seventy-nine percent of the coding sequences were transcribed in the same orientation as DNA replication, a feature that appears to be common in other low GC Gram-positive bacteria [15]. The replication termination site is localized near 1.1 megabase pairs by GC skew analysis. This region is located almost exactly opposite the origin of replication on the chromosome (Figure 1). The genome includes 65 pseudogenes, the majority of which are IS elements and hypothetical proteins.

Previous studies showed that the *S. pneumoniae *genome is rich in IS elements, which make up more of the genome than of any other bacterial genomes sequenced to date [13,15]. In the CGSP14 genome we identified 80 IS elements (Additional file 1). The majority of the IS elements appeared to be degenerate due to insertions, deletions, or point mutations, and only twelve were intact in CGSP14 genome. Although these degenerate IS elements might be inactive and non-functional, they could provide the potential sites for homologous recombination to acquire novel genes from related species.

### Comparative genomic analysis

**Figure 2:**
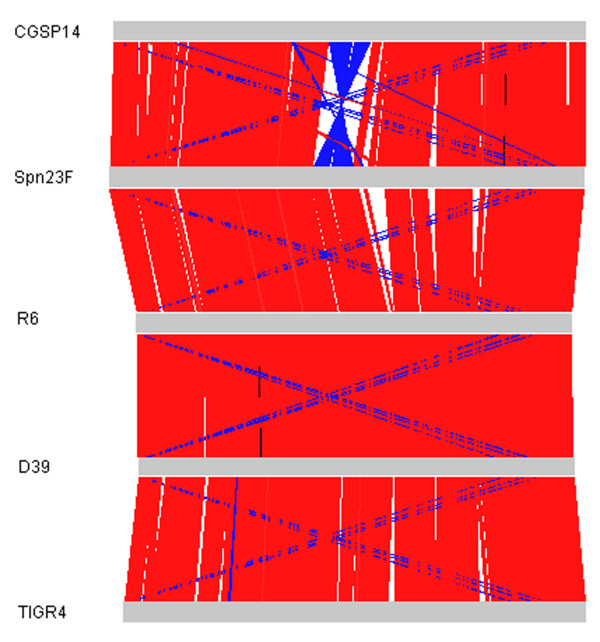
**Co-linearity comparison of five complete *S. pneumoniae *genomes based on BLAST and ACT**. Red lines indicate BLAST matches between genomes in the same direction; blue lines indicate BLAST matches between genomes in opposite directions.

Comparative analysis of CGSP14 genome with four complete genomes and twelve draft pneumococcal genomes (Table 1) provided new insights into the rapid evolution of the pneumococcal genome. *S. pneumoniae*, as with other bacterial pathogens, possesses a conserved core genome with interspersing regions of small and large scale differences (Figure 2). In total, 1,619 orthologous genes were shared by the seventeen pneumococcal genomes (Additional file 2). By searching the orthologous genes against COG database, 36% were found to be metabolism-related, 36% were associated with other known functions, 16% had poorly characterized functions, and 12% had no hits in the COG database and encoded mainly hypothetical proteins (Additional file 3). In addition, we found CGSP14 shared the largest number of orthologous genes (2055 and 2049 respectively) with strains Spn23F and SPnINV200 among the sixteen strains used for comparative genomic analysis, indicating that the CGSP14 genome shows highest homology to the two sequenced strains.

**Table 1:** Sixteen published genomes of *S. pneumoniae *used for comparative genomic analysis

Strain	Accession number	Serotype	Sequencing center
TIGR4	AE005672	4	TIGR
R6	AE007317	2	Eli Lilly and Company
D39	CP000410	2	TIGR
SP3-BS71	AAZZ00000000	3	Center for Genomic Sciences
SP6-BS73	ABAA00000000	6	Center for Genomic Sciences
SP9-BS68	ABAB00000000	9	Center for Genomic Sciences
SP11-BS70	ABAC00000000	11	Center for Genomic Sciences
SP14-BS69	ABAD00000000	14	Center for Genomic Sciences
SP18-BS74	ABAE00000000	6	Center for Genomic Sciences
SP19-BS75	ABAF00000000	19	Center for Genomic Sciences
SP23-BS72	ABAG00000000	23	Center for Genomic Sciences
G54	CP001015	19F	TIGR
Spn23F	FM211187	23F	Sanger
SPnINV104B	-	1	Sanger
SPnINV200	-	14	Sanger
SPnOXC141	-	3	Sanger

**Figure 3:**
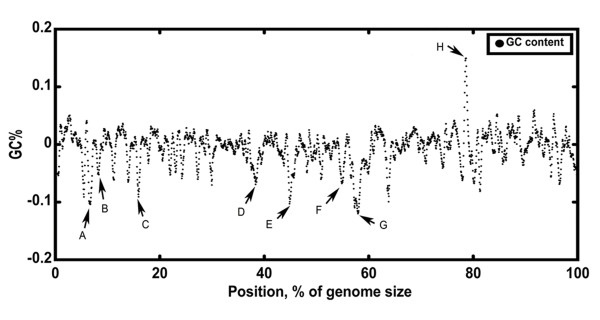
**GC content of the CGSP14 chromosome**. The GC content is calculated using the sliding-window technique (window size = 10 000 bp; shift size = 1 000 bp). Zero of the Y axis indicates the average GC content of the genome, which is 39.5%. Black arrows indicate transposons and potential genomic islands in the *S. pneumoniae *genome. A, lantibiotic synthesis gene cluster; B, Tn*3872*-like transposon; C, capsular locus; D, 19 kb inversion; E, possible phage remnant; F, putative resistance island; G, Tn*2008*; H, cell wall surface anchor family protein.

The genes on the distributed genomes were further analyzed. Alignment analysis revealed that at least eight distributed clusters were present in CGSP14 genome; most of the genes were related to virulence or antimicrobial resistance. These include a lantibiotic synthesis gene cluster, the capsular locus, a large cell wall surface anchor protein, two transposons, a resistance island, a possible phage remnant and a gene cluster with unknown functions. Meanwhile, we displayed the genome-wide GC content in Figure 3. All the eight clusters had deviated GC content, suggesting they could be recent acquisitions through horizontal gene transfer (HGT) in CGSP14. The distribution of the eight gene clusters among the seventeen pneumococcal genomes was shown in Table 2. Particularly, the two conjugative transposons were found to be unique in the CGSP14 genome.

**Table 2:** Distribution of the eight gene clusters among *S. pneumoniae *and other species of *Streptococcus*

Gene clusters	Strains of *S. pneumoniae*	Strains of other species (identity)
Lantibiotic biosynthesis proteins	CGSP14, SPnINV200	*S. thermophilus *CNRZ1066 (70–88%)
Tn*3872*-like transposon	CGSP14	-
Capsular locus	CGSP14, SP14-BS69, SPnINV200	-
19-kb insertion	CGSP14, SPnINV104B, SPnINV200, G54, TIGR4	-
Possible phage remnant	CGSP14, SP6-BS73, SP11-BS70, SP14-BS69, SP19-BS75, G54	*S. pyogenes *MGAS6180 (76–88%)
Putative resistance island	CGSP14, SP9-BS68, SP14-BS69, SP19-BS75, SPnINV200	-
Tn*2008*	CGSP14	-
Cell wall surface anchor protein	CGSP14, TIGR4	-

Alignment analysis indicated that chromosomal rearrangements occurred in *S. pneumoniae *(Figure 2). Compared with other published pneumococcal genomes, chromosomal inversions were identified in CGSP14 genome. A 189-kb inversion occurred across the replication termination site (from 1,010 kb to 1,199 kb). Chromosomal inversion across the replication axis usually is believed to rebalance the unbalanced chromosomal architecture caused by the insertion of large DNA segments [19]. In the CGSP14 genome, we found that most of the acquired-DNA segments (totally 80.5 kb), mainly composed of transposons and IS elements, resided in left of the replication axis (Figure 1). These observations suggested that the integration of transposons and IS elements affected the balance of the chromosomal architecture. This imbalance might cause the chromosomal inversion in CGSP14. This inversion led to transfer of 25 genes from the left to the right of the replication axis. Besides, a 19-kb inversion (from 832 kb to 851 kb) was observed in CGSP14 relative to TIGR4, G54, and INV200, while the gene order in this 19-kb segment is consistent to INV200. The gene cluster is not intact in other pneumococcal genomes. Further analysis showed that the four rearrangement breakpoints were located within the IS elements. Through chromosomal rearrangements, *S. pneumoniae *evolved to maintain genome stability after HGT that might confer genes necessary for the organism to survive or replicate in its environmental niche.

### Antimicrobial Resistance genes

CGSP14 is resistant to a variety of antimicrobial agents. The antimicrobial resistance determinants among the seventeen penumococcal strains were compared and listed in Additional file 4. CGSP14 contained 18 antimicrobial resistance determinants, while the number of antimicrobial resistance determinants in other strains varied from 9 to 12. Nearly half of the antimicrobial resistance determinants in CGSP14 were associated with mobile genetic elements.

**Figure 4:**
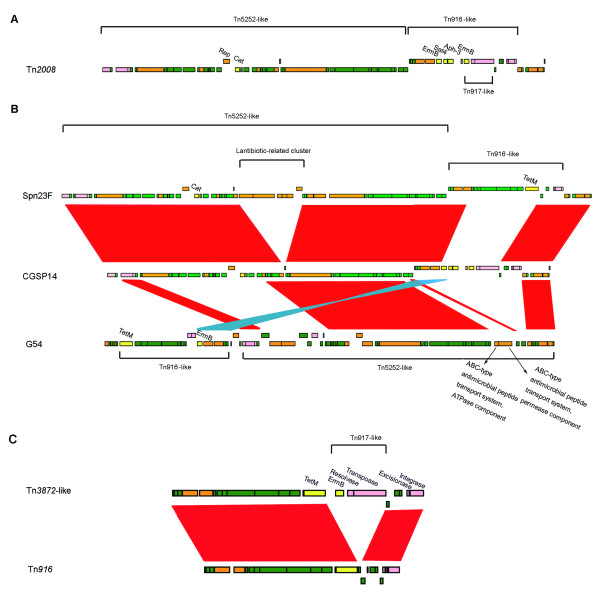
**Representation of the two conjugative transpsons in CGSP14**. (A) Schematic portrayal of the 68-kb transposon in CGSP14. Genes are coloured according to their putative functions. Pink boxes represent genes associated with mobility. Yellow boxes represent antimicrobial resistance genes. Orange boxes represent genes with other known functions. Green boxes represent hypothetical proteins. (B) Comparison of the conjugative transposons in the G54 and Spn23F genomes with CGSP14. Red bands indicate BLAST matches between genomes in the same direction; blue bands indicate BLAST matches between genomes in opposite directions. (C) Schematic portrayal of the Tn*3872*-like transposon in CGSP14 and comparison of this conjugative transposon with Tn*916*. The genes, *tetM*, *ermB*, *cat*, *sat*, and *aph-3*, confer tetracycline and minocycline resistance, erythromycin resistance, chloramphenicol resistance, streptothricin resistance, and kanamycin resistance, respectively.

The genome contained two large conjugative transoposons, which were found as composite elements of the known transposons. The first one containing 69 open reading frames (ORFs), was a 68-kb conjugative transposon (Figure 4A). Since this transposon had never been described previously, we named it Tn*2008*, a novel conjugative transposon. Sequence analysis indicated that Tn*2008 *was a composite of three transposons. A 50-kb DNA segment carrying chloramphenicol resistance gene (*cat*) could be an independent conjugative transposon and at left terminus of this transposon, two ORFs were designated as intergrase and relaxase required for transposition. The sequences of the ORFs within this transposon were highly homologous to those of Tn*5252*, which have been reported in *S. pneumoniae *before [20,21]. The Tn*5252*-like transposon was split into a 46-kb proximal region and a 4-kb distal region after the insertion of a 13-kb segment. The insertion appeared in the same position in Spn23F, which contained a 81-kb conjugative transposon [12]. The 13-kb insertion was identified as another independent transposon, which also owned the intergrase and excisionase at the right terminus for independent transposition; this transposon carried 3 genes coding for erythromycin, streptothricin and kanamycin resistance, a feature similar to the known Tn*1545 *[22]. Another 5-kb segment, as an insertion in the 13-kb transposon, resembled the transposon Tn*917 *[23], which contained 3 ORFs, encoding erythromycin resistance protein (*ermB*), resolvase and transposase. Overall, this novel conjugative transposon, a composite of three transposons, carried 5 antimicrobial resistance genes.

The other 23-kb conjugative transposon in CGSP14 contained 23 ORFs (Figure 4C). Sequence analysis demonstrated that this transposon was also a composite of two transposons. An 18-kb DNA segment carrying a tetracycline resistance gene (*tetM*) could be an independent transposon, which contained two ORFs encoding for intergrase and excisase; this transposon shows high similarity to the transposon Tn*916 *(Figure 4C) [24]. Another 5-kb segment carrying an erythromycin resistance gene (*ermB*), as an insertion, was identified as Tn*917*-like transposon. The structure of this composite transposon, i.e., a Tn*917*-like transposon inserted by a Tn*916 *transposon, resembled that of Tn*3872*. The structure of Tn*3872 *has been described in *S. pneumoniae *[25]; thus, this 23-kb conjugative transposon was defined as a Tn*3872*-like transposon.

Among the seventeen published *S. pneumoniae *genomes, an 81-kb conjugative transposon and a 67-kb conjugative transposon also appeared in the genomes of Spn23F and G54, respectively. The two conjugative transposons were both composed of a Tn*916*-like transposon and a Tn*5252*-like transposon [12,16], similar to Tn*2008 *in CGSP14. However, comparative analysis suggested that genetic variations occured among the three conjugative transposons (Figure 4B). In CGSP14 and Spn23F, the Tn*5252*-like transposons carry a chloramphenicol resistance gene, which seems missing in G54, and is replaced by an ABC-type antimicrobial peptide transport system. The Tn*916*-like transposon in Spn23F carries a tetracycline resistance gene, and in G54, it carries a tetracycline resistance gene and an erythromycin resistance gene. In contrast, the Tn*916*-like element of Tn*2008 *in CGSP14 lost the locus encoding the tetracycline resistance gene, while a DNA segment encoding three antimicrobial resistance genes, a transcriptional repressor and a Tn*917*-like transposon was inserted into this position. The variation of antimicrobial resistance determinants in the three conjugative transposons showed that the conjugative transposons have experienced frequent recombination and deletion events after the Tn*916*-like element integrated into the larger conjugative transposon, probably due to different selective pressures.

**Figure 5:**
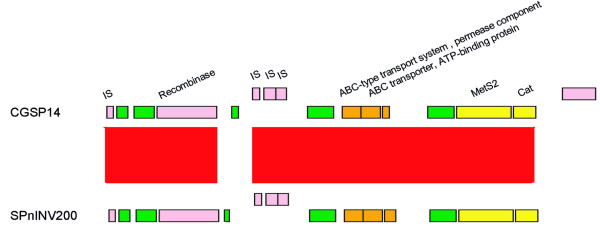
**Comparison of the putative resistance island in CGSP14 and SPnINV200**. Genes are coloured as in Figure 4. Red bands indicate BLAST matches between genomes in the same direction.*Cat *confers chloramphenicol resistance. *MetS2 *encodes methionyl-tRNA synthetase 2 which confers high-level MetS inhibitors resistance.

In addition to the two conjugative transposons carrying antimicrobial resistance genes, we identified a 14.4-kb genomic region (Figure 5), which appeared to be a resistance island in CGSP14. The 14.4-kb region carried a chloramphenicol resistance gene (*cat*) and a gene encoding methionyl-tRNA synthetase 2 (*metS2*). Further analysis showed that the island shared an average G+C content of 33.6%, much lower than the average of the genome (39.5%). This island contained several genes associated with genome instability, including one site-specific recombinase and multiple IS elements which might be responsible for the lateral transfer of the genomic region. Furthermore, the associated ORFs had diverse phylogenetic origin (data not shown). Based on these features, we deemed the 14.4-kb region as a resistance island; to our knowledge, this was for the first time described in *S. pneumoniae*. This 14.4-kb resistance island was also seen in the draft genomes of CGSSp14BS69, CGSSp19BS75, CGSSp9BS68 and SPnINV200. BLAST results showed that the sequences in this island showed high identity to each other. The comparison between CGSP14 and SPnINV200 was demonstrated in Figure 4. Since this island carried two antimicrobial resistance genes, the presence of this resistance island may be associated with the increased multidrug resistance of these strains.

Distinct from the characterized antimicrobial resistance determinants associated with mobile genetic elements in CGSP14, there were several chromosome-encoded determinants that also contributed to antimicrobial resistance. These included a tellurite resistance protein (*tehB*), a bacitracin resistance protein (*bacA*), a cadmium resistance transporter (*cadD*), a multidrug resistance efflux pump (*mdtG*), two β-lactam resistance factors (*femAB*), and three metallo-β-lactamases.

### Penicillin-binding proteins

Penicillin-resistant pneumococci are prevalent throughout the world. One mechanism conferring penicillin nonsusceptibility is alterations of penicillin-binding proteins (PBP). Alterations in PBP genes result in reduced affinity for penicillin and other β-lactams. Five high-molecular-weight PBP genes (*pbp1a, pbp1b, pbp2a, pbp2b, pbp2x*) dispersed in *S. pneumoniae*. Among the five genes, highly variable *pbp2x*, *pbp1a*, and *pbp2b *are considered most important in antimicrobial resistance [26]. By allele assignments of the three PBP genes in the seventeen penumococcal genomes, CGSP14 had the most variable *pbp2x*, *pbp1a*, and *pbp2b*. Although the sequence variations of these PBP genes in CGSP14 differed from two other strains of serotype 14 (SPnINV200 and SP14-BS69), they were almost identical to those of the strain Spn23F, suggesting that *pbp2x*, *pbp1a*, and *pbp2b *genes probably had gone through frequent homologous recombination between serotypes 14 and 23F.

### Virulence genes

The polysaccharide capsule is the principal pneumococcal virulence determinant. *S. pneumoniae *are divided into 91 serotypes depending on different capsular structures. Studies suggested that certain serotypes have a greater potential to cause invasive disease than others [27,28]. The clinical isolates of *S. pneumoniae *in Asia are largely confined to a limited number of serotypes, namely 6B, 9V, 14, 19F, and 23F [29]. In CGSP14 genome, a 19.4-kb gene cluster (SPCG0345 to SPCG0363) was identified to be involved in the synthesis of the capsular polysaccharide, flanked by two IS elements on each side, either truncated or disrupted, which were remnants of IS*1202 *and IS*1167*, respectively. Compared to strain 34359 of serotype 14 and strain SPnINV200 for which the capsular locus was determined [30], the capsular locus of CGSP14 differed at 3' end (Additional file 5). The gene *wciY *was divided into two *orf*s (SPCG0358 and SPCG0359) in CGSP14. This gene was unique in serotype 14, but its function was unknown. However, a previous study showed that the disruption of this gene did not affect capsular production [31]. Besides, the *orf *(SPCG0360) immediately downstream of these two genes in CGSP14 was found to contain a deletion of 5 units of a 306-bp tandem repeat, compared with the corresponding genes in strain 34359 and SPnINV200. The gene belonged to the surface anchored protein family but its function also remained unclear. With the exception of these two genes, other genes in the capsular locus were almost identical among the three strains of serotype 14 [30]. As has been described [30], serotype 14 utilized the Wzx/Wzy-dependent pathway to synthesize their capsular polysaccharide (Additional file 6).

In addition to the capsule, *S. pneumoniae *produced a number of other virulence factors, such as pneumolysin, hydrogen peroxide and cell surface proteins [32]. According to how they are linked to the cell surface, the surface proteins of *S. pneumoniae *are divided into three families: choline-binding proteins, LPXTG-anchored proteins, and lipoproteins [32]. Surface proteins of CGSP14 based on computer prediction are shown in Additional file 7.

Several members of the choline-binding protein family are known to be important for virulence, including the autolysin (*lytA*), choline binding protein A (*pspC*), and pneumococcal surface protein A (*pspA*). PspC is involved in the adhesion of bacteria to the nasopharynx [33]. PspA is a highly variable protein and involved in inhibition of complement activation [32]. Choline binding protein PcpA is postulated to be an adhesin because it contains leucine-rich repeats [34]. The seventeen penumococcal genomes all harbored one copy of these virulence determinants, while CGSP14 and SP19-BS75 both obtained another copy of *pspA *and *pcpA *due to a 7-kb-long DNA insertion adjacent to a remnant transposase. The 7 kb sequences in CGSP14 and SP19-BS75 showed high identity to each other.

Proteins that contain the LPXTG amino acid motif are common in most Gram-positive bacteria. The LPXTG motif near to the carboxyl terminal of the protein is recognized and linked to the cell wall by a sortase enzyme [32]. Neuraminidase is one of the LPXTG-anchored proteins. Neuraminidase cleaves N-acetylneuraminic acid from oligosaccharides, glycoproteins, glycolipids and is viewed as a virulence factor in microbial pathogenesis [32]. Analysis of the available genome sequences of *S. pneumoniae *indicated that this microorganism had at least three neuraminidases [13-15]. All the three neuraminidases are present in CGSP14. Both *nanA *and *nanB *are present in all the other sixteen penumococcal strains, while *nanC *is present only in eight. The presence of *nanC *might be associated with the increased virulence of some strains of *S. pneumoniae*. Zinc metalloprotease is also a member of LPXTG-anchored protein family. From the published genome sequences of *S. pneumoniae*, four zinc metalloproteases were discovered. CGSP14 contained three of them, including *iga*, *zmpB *and *zmpD*. Zinc metalloproteinases belong to a group of hypervariable surface proteins, the hypervariability of these proteins are due to frequent HGT in these regions, enabling antigenic escape [35].

Besides these common virulence proteins, one unusual protein in LPXTG-anchored proteins family was found. The gene, SPCG1750, encoded a 4695-animo acid protein, containing 528 imperfect repeats of the amino acid motif SASASAST. This surface protein shows homology to SP1772 (4776-animo acid) in TIGR4. The surface protein is located in the vicinity of nine glycosyl transferases in CGSP14, all of which are present on a 40.5-kb segment flanked by two IS elements. The 40.5-kb region seems to be an insertion in CGSP14 and TIGR4 due to HGT, as this region has not been found in other genomes.

**Figure 6:**
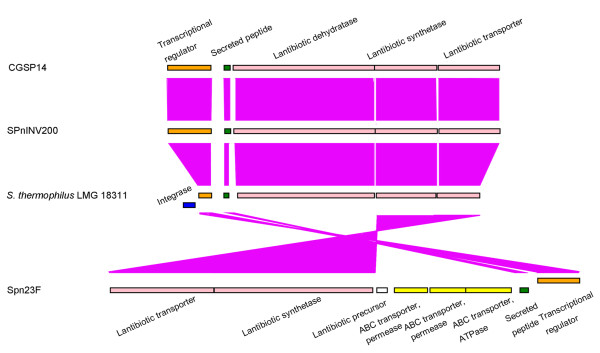
**Comparison of the lantibiotic synthesis loci of CGSP14 and other genomes**. Genes are represented by boxes coloured according to the gene key, with gene designations above or below each box. The genes encoded in this locus in the genomes of CGSP14, SPnINV200 and *S. thermophilus *are highly homologous, but they have no sequence similarity to those in the genome of SPN23F. Purple bands correspond to genes that have similar function in the lantibiotic synthesis in each genome.

Lantibiotics are peptide antibiotics with high antimicrobial activity against several Gram-positive bacteria. They are ribosomally synthesized and posttranslationally modified [36]. In CGSP14, we identified a 5.4-kb locus encoding three proteins related to lantibiotic biosynthesis: a lantibiotic dehydratase, a lantibiotic synthetase and a lantibiotic efflux protein, nearby a transcriptional regulator (Figure 6). By a thorough search against other sixteen genomes, this gene cluster shows high similarity to the corresponding locus in the genome of another serotype 14 strain SPnINV200. Recent studies reported that the strains SP23-BS72 and Spn23F of serotype 23 also contained lantibiotic synthesis gene clusters [12,37]; however, comparative analysis indicated that they showed no sequence similarity to those found in CGSP14 and SPnINV200. Furthermore, we performed a BLAST search against the nr database, and found the locus in the serotype14 has a high similarity (70%–88% identity) to that in *Streptococcus thermophilus*. Therefore, this locus in the serotype 14 might encode a new type of lantibiotic, different from those found in the serotype 23. This finding suggests that communication of virulence genes has occurred among different species of *Streptococci*.

### What drives the genome evolution?

In this study, we further analyzed 20 clinical isolates of *S. pneumoniae *serotype 14, all from sterile sites, by multilocus sequence typing (MLST), in addition to CGSP14, which belonged to ST15. The most common sequence type was ST876 (7 isolates), followed by ST13 (5) and ST46 (5) (Table 2). Only one ST15 was identified among the 20 isolates. ST876 and ST46 were prevalent in Taiwan, while both ST13 and ST15 belonged to variants of the international England^14 ^clone (ST9). Capsular switching could have occurred between serotypes 14 and 3 and between serotype 14 and serogroup 9, as we found two isolates (Bsp097 and Bsp098) were ST1569. ST1569 has been identified in serotypes 3, 9A, and 9V. All the clinical serotype 14 isolates, expressed different levels of penicillin and ceftriaxone nonsusceptibility (Table 3), which is in accord with the data published recently [38]. This finding indicates that higher competence and plasticity of the genome likely afforded an advantage to pneumococcal strains to become more and more antimicrobial-resistant, and again supports that virulent clones might evolve to be more resistant in order to survive in the drug environment. Given the fact, to reduce the selective pressure, judicious use of antibiotics should never be overemphasized.

**Table 3:** Multilocus sequence typing and antimicrobial susceptibility of *S. pneumoniae *serotype 14 isolates

		MLST allelic profile							MIC (μg/ml)	
		
Strain	Sequence type (corresponding serotypes)	*aroE*	*gdh*	*gki*	*recP*	*spi*	*xpt*	*ddl*	Penicillin	Ceftriaxone
CGSP14	15 (14)	1	5	4	5	5	3	8	2	1
SP0153	876 (14)	8	13	14	4	4	6	14	4	1
SP0166	876 (14)	8	13	14	4	4	6	14	4	0.5
SP0286	876 (14)	8	13	14	4	4	6	14	8	1
SP0336	876 (14)	8	13	14	4	4	6	14	0.5	0.5
SP0340	876 (14)	8	13	14	4	4	6	14	4	2
SP0363	876 (14)	8	13	14	4	4	6	14	4	1
SP0386	876 (14)	8	13	14	4	4	6	14	4	1
SP0210	13 (14)	1	5	4	5	5	27	8	8	2
SP0226	13 (14)	1	5	4	5	5	27	8	4	1
SP0233	13 (14)	1	5	4	5	5	27	8	4	1
SP0234	13 (14)	1	5	4	5	5	27	8	6	2
SP0282	13 (14)	1	5	4	5	5	27	8	4	1
Ssp017	46 (14)	1	5	4	16	31	1	14	0.5	8
Ssp097	46 (14)	1	5	4	16	31	1	14	0.5	0.25
SP017	46 (14)	1	5	4	16	31	1	14	0.5	0.5
SP044	46 (14)	1	5	4	16	31	1	14	8	2
SP033	46 (14)	1	5	4	16	31	1	14	8	8
Bsp097	1569 (9V, 3, 9A)	8	11	10	1	6	8	1	4	2
Bsp098	1569 (9V, 3, 9A)	8	11	10	1	6	8	1	4	4
SP0232	15 (14)	1	5	4	5	5	3	8	0.25	0.5

## Discussion

A bacterial pathogen can be described by its "supragenome", which is composed of a "core genome" and an "distributed genome" [39-41]. In general, the core genome includes all genes responsible for the basic aspects of the biology of a species and its major phenotypic traits. In contrast, distributed genomes constitute to the species diversity and might encode supplementary biochemical pathways and functions not essential for bacterial growth but which confer selective advantages, such as adaptation to different niches and antimicrobial resistance. In this study, we added a complete genome to the pneumococcal supragenome. The analysis of distributed genome of CGSP14 indicates that pneumococcal supragenome is still open and evolving.

Comparative analysis showed that gene communications occurred frequently among different strains of *S. pneumoniae *and among different species of the streptococcus genus by HGT, which has been demonstrated as a major force to drive bacterial evolution [42,43]. *S. pneumoniae *is able to efficiently acquire genetic materials from the large gene pool of the environment by means of transformation, transduction, and conjugation. We found that at the genome level, CGSP14 acquired at least eight foreign DNA elements from other organisms by HGT, as these blocks demonstrated deviation in the GC content. In sequence analysis, the genes encoded in these newly acquired elements may enhance the pathogenicity and antimicrobial resistance of CGSP14.

In CGSP14 genome, a remarkable feature is horizontal acquisition of two conjugative transposons and one genomic island. The three previously un-described mobile elements totally carried eight antimicrobial resistance genes, which promote the adaptation of organism to an environment with high drug pressure. Besides, most of the other accessory DNA elements were flanked by IS elements; for instance, the capsular locus, the predominant virulence determinant of *S. pneumoniae*, was a mobile genetic element flanked by IS elements. The dynamics of these mobile elements might attribute to the clinically important phenotypic shift in *S. pneumonae*.

Invasive pneumococcal disease cases due to serotypes included in the 7-valent vaccine continued to fall in the USA, but the overall invasive pneumococcal disease rate leveled off starting in 2002, largely due to an increase in cases caused by serotype 19A, which is not covered by the 7-valent vaccine [44]. Given these trends, development of expanded-valency polysaccharide vaccines or ideally, a universal protein vaccine, is mandatory. It has been shown that in the case of *S. agalactiae*, the design of such vaccine was only possible using virulence-related dispensable genes [44]. To this end, sequencing of multiple genomes from *S. pneumoniae *to better probe the diversity of the pathogen and its pathogenic features is necessary and certainly will continue to surprise us with fascinating discoveries in the evolution of *S. pneumoniae *and impact on the clinical medicine.

## Conclusion

*Streptococcus pneumoniae *serotype 14 is one of the most invasive among > 90 pneumococcal serotypes, often causing life-threatening invasive pneumococcal diseases in humans. *S. pneumoniae *has been evolving rapidly over time, resulting in a large amount of genetic diversity, despite the constraints imposed by the small genome size and complex genetic organization of the genome. In this study, we sequenced the entire genome of a serotype 14 clinical isolate (CGSP14), and carried out comprehensive comparison with other pneumococcal genomes. We found that the genome evolution of *S. pneumoniae *is driven largely by the host adaptations. In addition to horizontal gene transfer, recombination, which re-balanced the genome structure after events of gene loss or addition, also appears to be an important element in *S. pneumoniae *evolution. Human intervention in the form of mass vaccination and antimicrobial treatment reduced the burden of pneumococcal diseases, but has already accelerated the evolution of the pneumococcal genome. We conclude that such evolution results in a more virulent and antimicrobial-resistant *S. pneumoniae *serotype 14.

## Methods

### Bacterial strains

Twenty-one *S. pneumoniae *serotype 14 isolates, including the sequenced CGSP14, were collected from patients with bacteremic pneumonia treated in Chang Gung Memorial Hospital and Children's Hospital, Taoyuan, Taiwan between 2004 and 2005. Serotyping was carried out by the quellung reaction with antisera from the Statens Serum Institut, Copenhagen, Denmark and verified by sequential multiplex PCR [45]. Minimum inhibitory concentrations (MICs) were determined by the E test (AB Biodisk, Solna, Sweden). CGSP14 is a serotype 14 clinical isolate derived from a child with necrotizing pneumonia, simultaneously complicated with HUS. The MICs of antimicrobial agents to CGSP14 are penicillin, 2 μg/mL, erythromycin, 256 μg/mL, and ceftriaxone, 1 μg/mL.

### Multilocus sequence typing (MLST)

The nucleotide sequences of 450-bp internal regions from the *aroE*, *ddl*, *gdh*, *gki*, *recP*, *spi*, and *xpt *genes were amplified by PCR using the primers described previously [46]. The gene fragments were sequenced on both strands, by using the same primers, on an ABI 3730 automated sequencer (Applied Biosystems, Foster City, USA). The sequences were then compared with those of the recognized alleles of each gene listed in the pneumococcal MLST website database  by using BioEdit Sequence Alignment Editor. The web database  was used for assigning allele numbers for particular loci and the sequence type (ST) of each isolate was defined on the basis of the resulting allelic profiles.

### Whole genome sequencing

The whole genome of *S. pneumoniae *CGSP14 was sequenced by using the random shotgun method. Three genomic libraries (1.5~2-kb inserts, 2~3-kb inserts and 4-kb inserts) were constructed from randomly sheared genomic DNA. Random clones were sequenced using ET-Dye terminator chemistry, analyzed with an ABI 3700 sequencer (Applied Biosystems, Foster City, USA) and a MegaBACE 1000 sequencer (Amersham Biosciences, Sweden). DNA sequences were analyzed and assembled using PHRED, PHRAP, and CONSED [47-49]. Gaps in the sequence were closed either by sequencing through primer-walking the plasmid templates or by direct sequencing of combinatorial PCR products. The completed genome contained 34,888 reads with an average length of 501 bp, resulting in 8-fold sequence coverage. The complete genome sequence of CGSP14 has been deposited in the GenBank database with the accession number CP001033.

### Genome annotation

Open reading frames (ORFs) were predicted by GLIMMER with the default parameters [50]. Putative ORFs shorter than 30 amino acids were eliminated and ORFs that overlapped were visually inspected, and removed as needed. The predicted ORFs were reviewed to define start codons on the basis of ribosomal-binding motifs and homologies. ORFs were further searched against the non-redundant protein database using BLAST [50]. Functional domains of putative proteins were identified by searching against Prosite, Blocks, and Pfam database . Functional categories were assigned by searching all predicted proteins against COG database . Transfer RNA genes (tRNAs) were identified using tRNAscan-SE [51], ribosomal RNA genes (rRNAs) and other structural RNAs were identified from BLAST similarity searches.

### Comparative genomic analysis

Twelve penumococcal genome sequences and the protein-coding sequences per strain (R6: AE007317; D39: CP000410; TIGR4: AE005672; G54: CP001015; SP3-BS71: AAZZ00000000; SP6-BS73: ABAA00000000; SP9-BS68: ABAB00000000; SP11-BS70: ABAC00000000; SP14-BS69: ABAD00000000; SP18-BS74: ABAE00000000; SP19-BS75: ABAF00000000 and SP23-BS72: ABAG00000000) were obtained through the web site of NCBI . The remaining four genome sequences (Spn23F, SPnINV104B, SPnINV200, and SPnOXC141) were obtained through the web site of Sanger Institute , and the ORFs per strain were predicted by GLIMMER and annotated by searching against the nr database using BLAST. Each pair of the predicted ORFs among the seventeen penumococcal strains were aligned using BLASTP (E value < 10^-5 ^and protein similarity > 40%). The outputs were parsed by perl scripts written by ourselves to extract the orthologous genes shared among the seventeen penumococcal genomes. Alignment of the genomes of the five completely sequenced strains was accomplished with the MUMmer program [52]. ACT (Artemis Comparison Tool) was used to enable the visualization of BLAST comparisons between the genomes [53]. Single nucleotide polymorphism (SNP) analysis of each orthologous gene was accomplished using CLUSTALW [54].

## Abbreviations

MLST: multilocus sequence typing; HGT: horizontal gene transfer; HUS: hemolytic uremic syndrome; CDS: protein-coding sequence; ORF: open reading frame; ST: sequence type.

## Competing interests

The authors declare that they have no competing interests.

## Authors' contributions

CHC, PT, and JY conceived and designed the experiments. FD, MHH, and SH performed the experiments. FD, PT, PC, and SH analyzed the data. FD and CHC wrote the paper. All authors read and approved the final manuscript.

## Supplementary Material

Additional file 1 **IS elements of the CGSP14 genome**. The data show the list of the IS elements in the CGSP14 genome. Click here for file

Additional file 2 **Orthologues shared among the seventeen *S. pneumoniae *genomes**. The data represent the core genes of the seventeen *S. pneumoniae *genomes, assigned by their accession numbers in CGSP14 Click here for file

Additional file 3 **COG assignment of the orthologous genes**. The data show the alignment results by searching the core genes against COG database. Click here for file

Additional file 4 **Comparison of antimicrobial resistance determinants among the seventeen *S. pneumoniae *genomes**. The data show antimicrobial resistance determinants in the seventeen *S. pneumoniae *genomes. Click here for file

Additional file 5 **Capsule biosynthesis genes of CGSP14 and comparison of the capsular loci among three serotype 14 strains**. This figure shows capsule biosynthesis genes of *S. pneumoniae *serotype 14 strains. Genes are represented by boxes colored according to the gene key, with gene designations above or below each box. Red bands indicate regions that are highly homologous between gene clusters. Click here for file

Additional file 6 **Capsular biosynthesis pathway of *S. pneumoniae *CGSP14**. This figure shows the capsule biosynthesis pathway of *S. pneumoniae *CGSP14. Click here for file

Additional file 7 **Putative surface proteins based on computer prediction**. The data show the list of putative surface proteins of *S. pneumoniae *CGSP14. Click here for file
