# Supplementary material for: Genome evolution driven by host adaptations results in a more virulent and antimicrobial-resistant Streptococcus pneumoniae serotype 14
Source: BMC Genomics. 2009 Apr 13;10:158. doi: 10.1186/1471-2164-10-158 (PMC2678160; doi:10.1186/1471-2164-10-158)
Supplement: Additional file 1 — IS elements of the CGSP14 genome. The data show the list of the IS elements in the CGSP14 genome. [file 1471-2164-10-158-S1.doc]

**Additional file 1. IS elements of the CGSP14 genome.**

| IS family | Name | Length | Intact transposase | Truncated or frameshifted |
| --- | --- | --- | --- | --- |
| IS3 | IS3-Spn1 | 1414 | 0 | 6 |
| IS4 | IS4 | 1426 | 0 | 1 |
| IS5 | IS1381-Spn1 | 846 | 0 | 12 |
| IS5 | IS1515 | 861 | 0 | 1 |
| IS30 | IS1239 | 1080 | 4 | 1 |
| IS66 | IS66 | 2548 | 0 | 3 |
| IS110 | IS110 | Unknown | 0 | 3 |
| IS605 | IS200 | 747 | 0 | 1 |
| IS630 | IS630-Spn1 | 1153 | 0 | 9 |
| IS1380 | IS1380-Spn1 | 1665 | 4 | 1 |
| ISL3 | IS1167 | 1435 | 4 | 11 |
| ISNCY* | IS1202 | 3535 | 0 | 2 |
| Unknown |  |  | 0 | 17 |
| Total |  |  | 12 | 68 |

*ISNCY refers to unclassified IS elements.
