# Supplementary figures and images for: Genome evolution driven by host adaptations results in a more virulent and antimicrobial-resistant Streptococcus pneumoniae serotype 14
Source: BMC Genomics. 2009 Apr 13;10:158. doi: 10.1186/1471-2164-10-158 (PMC2678160; doi:10.1186/1471-2164-10-158)

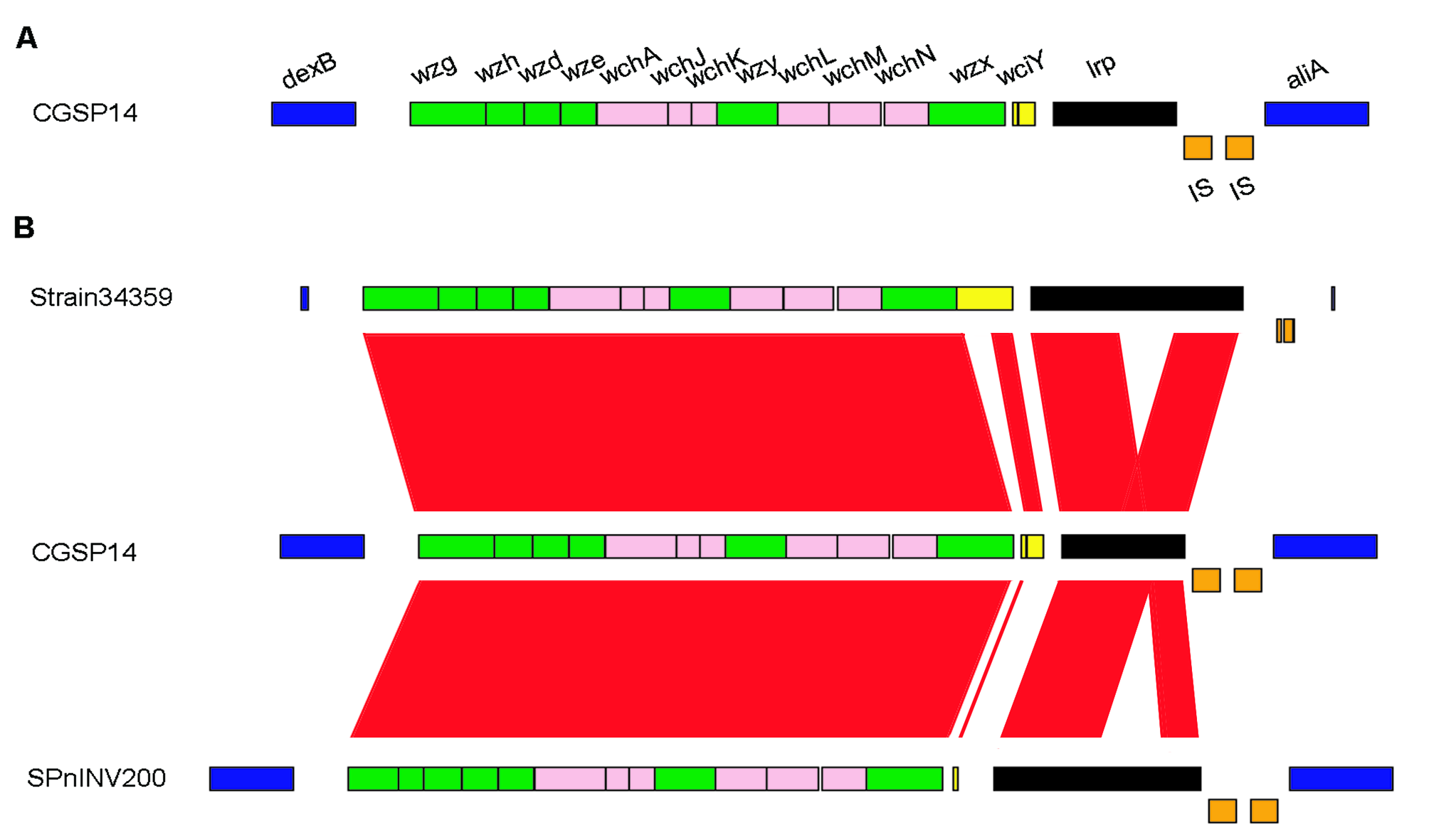

Supplement: Additional file 5 — Capsule biosynthesis genes of CGSP14 and comparison of the capsular loci among three serotype 14 strains. This figure shows capsule biosynthesis genes of S. pneumoniae serotype 14 strains. Genes are represented by boxes colored according to the gene key, with gene designations above or below each box. Red bands indicate regions that are highly homologous between gene clusters. [file 1471-2164-10-158-S5.tiff]

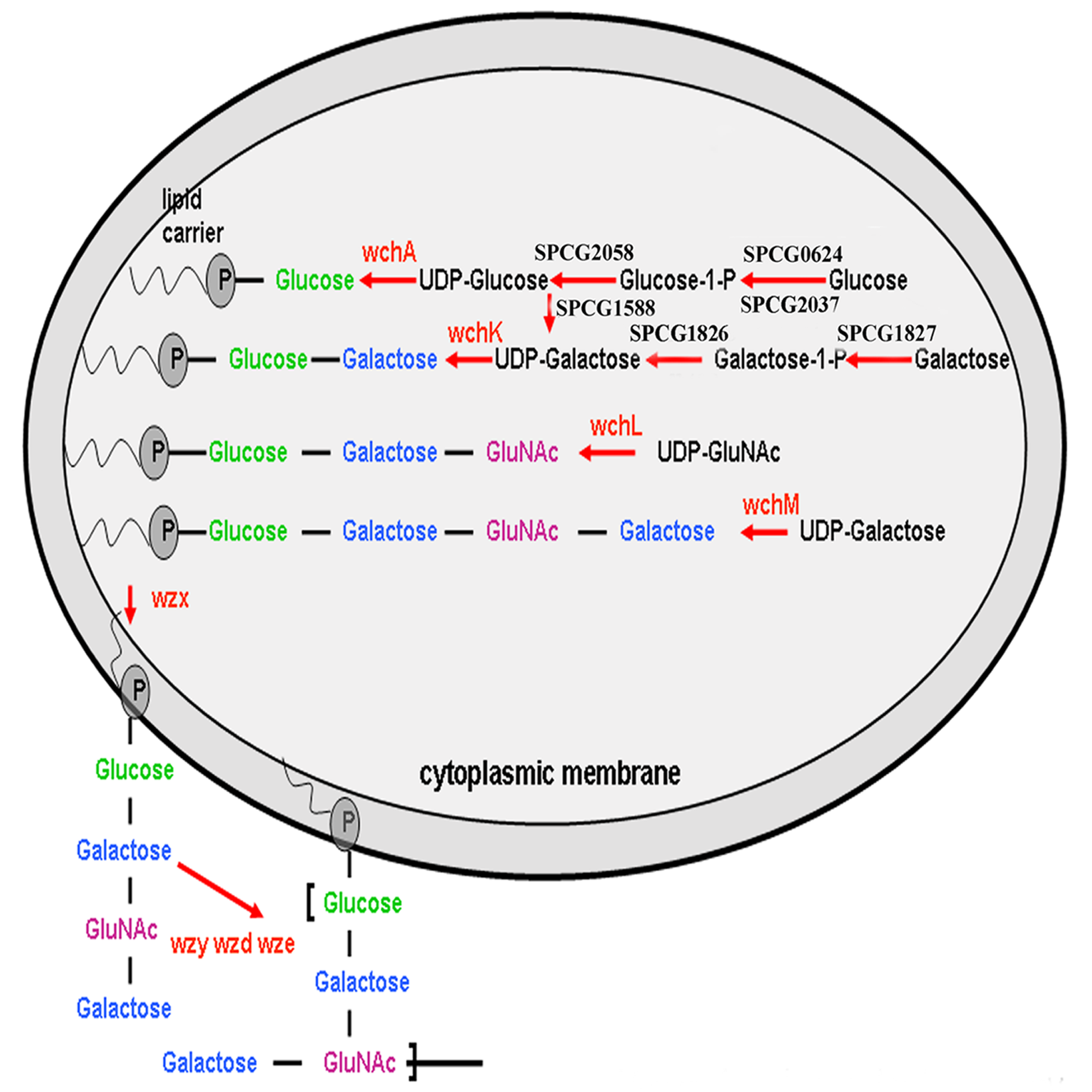

Supplement: Additional file 6 — Capsular biosynthesis pathway of S. pneumoniae CGSP14. This figure shows the capsule biosynthesis pathway of S. pneumoniae CGSP14. [file 1471-2164-10-158-S6.tiff]
