# Supplementary material for: Genome evolution driven by host adaptations results in a more virulent and antimicrobial-resistant Streptococcus pneumoniae serotype 14
Source: BMC Genomics. 2009 Apr 13;10:158. doi: 10.1186/1471-2164-10-158 (PMC2678160; doi:10.1186/1471-2164-10-158)
Supplement: Additional file 7 — Putative surface proteins based on computer prediction. The data show the list of putative surface proteins of S. pneumoniae CGSP14. [file 1471-2164-10-158-S7.doc]

**Additional data file** 7. Putative surface proteins based on computer prediction

| ORF | Description | LPXTG | Choline binding | Lipoprotein |
| --- | --- | --- | --- | --- |
| SPCG0278 | Alkaline amylopullulanase, putative | + |  |  |
| SPCG0603 | Beta-galactosidase (BgaA) | + |  |  |
| SPCG0059 | Beta-N-acetylhexosaminidase (StrH) | + |  |  |
| SPCG0080 | Cell wall surface anchor family protein | + |  |  |
| SPCG1750 | Cell wall surface anchor family protein | + |  |  |
| SPCG1811 | Cell wall surface anchor family protein | + |  |  |
| SPCG1957 | Cell wall surface anchor family protein | + |  |  |
| SPCG0364 | Cell wall surface anchor family protein,authentic frameshift | + |  |  |
| SPCG0476 | Endo-beta-N-acetylglucosaminidase, putative | + |  |  |
| SPCG0322 | Hyaluronidase | **+** |  |  |
| SPCG1143 | Immunoglobulin A1 protease (Iga) | + |  |  |
| SPCG0620 | Zinc metalloprotease ZmpB, putative | + |  |  |
| SPCG1142 | zinc metalloprotease ZmpD, putative | + |  |  |
| SPCG0599 | Serine protease, subtilase family | + |  |  |
| SPCG2105 | Choline-binding protein (PcpA) |  | + |  |
| SPCG1256 | Choline-binding protein (PcpA) |  | + |  |
| SPCG2158 | Choline-binding protein A (CbpA) |  | + |  |
| SPCG2168 | Choline-binding protein D (CbpD) |  | + |  |
| SPCG0905 | Choline-binding protein E (CbpE) |  | + |  |
| SPCG0387 | Choline-binding protein G (CbpG) |  | + |  |
| SPCG0373 | Choline-binding protein J (CbpJ) |  | + |  |
| SPCG1911 | Autolysin (LytA) |  | + |  |
| SPCG1559 | Lysozyme (LytC) |  | + |  |
| SPCG0941 | Endo-beta-N-acetylglucosaminidase (LytB) |  | + |  |
| SPCG1257 | Pneumococcal surface protein A (PspA) |  | + |  |
| SPCG0120 | Pneumococcal surface protein A (PspA) |  | + |  |
| SPCG0623 | Pneumococcal surface protein, putative |  | + |  |
| SPCG1665 | Neuraminidase A |  | + |  |
| SPCG1660 | Neuraminidase B |  | + |  |
| SPCG0853 | Neuraminidase C |  | + |  |
| SPCG1808 | ABC transporter, substrate-binding protein |  |  | + |
| SPCG0089 | ABC transporter, substrate-binding protein |  |  | + |
| SPCG1663 | ABC transporter, substrate-binding protein |  |  | + |
| SPCG1773 | ABC transporter, substrate-binding protein |  |  | + |
| SPCG2165 | ABC transporter, substrate-binding protein |  |  | + |
| SPCG0152 | ABC transproter, substrate-binding protein |  |  | + |
| SPCG1383 | Amino acid ABC transporter, amino acid binding protein |  |  | + |
| SPCG1485 | Amino acid ABC transporter, amino acid binding protein |  |  | + |
| SPCG0108 | Amino acid ABC transporter, periplasmic amino acid binding protein |  |  | + |
| SPCG0581 | Amino acid ABC transproter, amino acid binding protein, putative |  |  | + |
| SPCG1009 | Iron-compound ABC transporter, iron compound-binding protein |  |  | + |
| SPCG1845 | Iron-compound ABC transporter, ironcompound binding protein |  |  | + |
| SPCG2072 | Maltose/maltodextrin ABC transporter,maltose/maltodextrin-binding protein (MaIX) |  |  | + |
| SPCG1623 | Manganese ABC transporter, manganesebinding adhesion liprotein |  |  | + |
| SPCG1514 | Oligopeptide ABC transporter, oligopeptidebinding protein (AliB) |  |  | + |
| SPCG1865 | Oligopeptide ABC transporter, oligopeptidebinding protein (AmiA) |  |  | + |
| SPCG2050 | Phosphate ABC transporter, phosphatebinding protein (PstS) |  |  | + |
| SPCG1389 | Phosphate ABC transporter, phosphatebinding protein, putative |  |  | + |
| SPCG1656 | Sugar ABC transporter, sugar-binding protein |  |  | + |
| SPCG1871 | Sugar ABC transporter, sugar-binding protein (MsmE) |  |  | + |
| SPCG2137 | Zinc ABC transporter, zinc-binding lipoprotein (AdcA) |  |  | + |
| SPCG0699 | Branched-chain amino acid ABC transporter, amino acid-binding protein |  |  | + |
| SPCG1062 | Amino acid ABC transporter, amino acid-binding protein/permease protein |  |  | + |
| SPCG0448 | Amino acid ABC transporter, amino acid-binding protein/permease protein |  |  | + |
| SPCG0570 | Amino acid ABC transporter, amino acid-binding protein |  |  | + |
| SPCG0658 | Amino acid (glutamine) ABC transporter substrate binding protein |  |  | + |
| SPCG0363 | Oligopeptide ABC transporter, oligopeptide-binding protein AliA |  |  | + |
| SPCG1374 | Spermidine/putrescine ABC transporter, spermidine/putrescine-binding protein |  |  | + |
| SPCG2134 | Probable sugar ABC transporter, sugar-binding protein |  |  | + |
| SPCG0153 | Lipoprotein |  |  | + |
| SPCG0787 | Lipoprotein |  |  | + |
| SPCG0976 | Adhesion lipoprotein |  |  | + |
| SPCG0590 | Conserved hypothetical protein |  |  | + |
| SPCG0875 | Conserved hypothetical protein |  |  | + |
| SPCG0204 | Hypothetical protein |  |  | + |
| SPCG0209 | Hypothetical protein |  |  | + |
| SPCG0720 | Peptidyl-prolyl cis-trans isomerase, cyclophilin-type |  |  | + |
| SPCG0956 | Protease maturation protein, putative |  |  | + |
| SPCG1939 | OxaA-like protein precursor |  |  | + |
| SPCG2007 | SpoIIIJ family protein |  |  | + |
| SPCG0615 | Thioredoxin family protein |  |  | + |
| SPCG0974 | Thioredoxin family protein |  |  | + |
